# Identification and Analysis of microRNAs in *Chlorella sorokiniana* Using High-Throughput Sequencing

**DOI:** 10.3390/genes11101131

**Published:** 2020-09-25

**Authors:** Siti Nor Ani Azaman, Dilan Amila Satharasinghe, Sheau Wei Tan, Norio Nagao, Fatimah M. Yusoff, Swee Keong Yeap

**Affiliations:** 1 Centre of Foundation Studies for Agricultural Science, Universiti Putra Malaysia, Serdang 43400, Selangor, Malaysia; s_norani@upm.edu.my; 2 Aquatic Animal Health and Therapeutics Laboratory (AquaHealth), Institute of Bioscience, Universiti Putra Malaysia, Serdang 43400, Selangor, Malaysia; 3 Department of Basic Veterinary Sciences, Faculty of Veterinary Medicine and Animal Science University of Peradeniya, Peradeniya 20400, Sri Lanka; das@pdn.ac.lk; 4 Laboratory of Vaccine and Biomolecules (VacBio), Institute of Bioscience, Universiti Putra Malaysia, Serdang 43400, Selangor, Malaysia; tansheau@upm.edu.my; 5 102 Naname-go, Shinkamigoto-cho, Minami Matsuura-gun, Nagasaki 857-4214, Japan; norio.nagao@upm.edu.my; 6 Department of Aquaculture, Faculty of Agriculture, Universiti Putra Malaysia, Serdang, Selangor 43400, Malaysia; fatimamy@upm.edu.my; 7 China-ASEAN College of Marine Sciences, Xiamen University Malaysia, Sepang, 43900 Selangor, Malaysia

**Keywords:** *Chlorella sorokiniana*, miRNA, metabolites, transcription regulation

## Abstract

*Chlorella* is a popular microalga with robust physiological and biochemical characteristics, which can be cultured under various conditions. The exploration of the small RNA content of *Chlorella* could improve strategies for the enhancement of metabolite production from this microalga. In this study, stress was introduced to the *Chlorella sorokiniana* culture to produce high-value metabolites such as carotenoids and phenolic content. The small RNA transcriptome of *C. sorokiniana* was sequenced, focusing on microRNA (miRNA) content. From the analysis, 98 miRNAs were identified in cultures subjected to normal and stress conditions. The functional analysis result showed that the miRNA targets found were most often involved in the biosynthesis of secondary metabolites, followed by protein metabolism, cell cycle, and porphyrin and chlorophyll metabolism. Furthermore, the biosynthesis of secondary metabolites such as carotenoids, terpenoids, and lipids was found mostly in stress conditions. These results may help to improve our understanding of regulatory mechanisms of miRNA in the biological and metabolic process of *Chlorella* species. It is important and timely to determine the true potential of this microalga species and to support the potential for genetic engineering of microalgae as they receive increasing focus for their development as an alternative source of biofuel, food, and health supplements.

## 1. Introduction

*Chlorella* is known as one of the best microalgae for the production of high-value metabolites such as carotenoids and lipids [1,2]. Microscopic observation of lipid-droplet and starch-granule accumulation in algal cells proved the potential of *Chlorella* as an alternative source for biofuel production [3]. Several researchers reported that the manipulation of different environmental factors could affect biochemical production from microalgae [4,5]. For instance, *Chlorella sorokiniana* showed a several fold increase in carotenoid production when cultured under stress-related conditions, regulated through a carotenoid biosynthesis pathway [6].

Although metabolic transition was identified in the accumulation of pigment, lipid, and starch in *Chlorella* species [7], there is still a lack of genetic information and differential expression of key metabolic pathways that hamper manipulation and improvement of these microalgae. This suggests that the factors and mechanisms responsible for the differences in metabolite deposition, which have not been elucidated completely, could aid in the development of new strategies to modulate carotenoid and lipid deposition and improve their commercial value.

Small noncoding RNA (ncRNA) genes have been found in numerous organisms, acting as key regulators for development through modulation of the processing, stability, and translation of larger RNAs. Their ability to silence specific genes affects a wide range of biological functions, ranging from gene regulation during embryological development and cell differentiation to genome rearrangement [8,9]. Of the classes of small RNAs, microRNAs (miRNAs) play a pivotal role in controlling gene expressions that are involved in developmental, physiological, or metabolic processes, or stress responses through messenger RNA (mRNA) cleavage or translational repression [10,11]. The choice of control mechanism by miRNA is dependent on the degree of sequence complementary between miRNAs and their target, in which perfect pairing results in target mRNA cleavage, whereas imperfect pairing results in repression. The other mechanism of miRNA-mediated regulation is miRNA-mediated mRNA decay [12,13].

This study is the first step toward profiling the miRNAs that are involved in regulating the production of carotenoids and lipids in *Chlorella sorokiniana* when cultured under moderate light intensity and glucose addition during nitrogen-limited conditions using a small RNA-sequencing technique. This in turn may elucidate the important metabolic pathways (involving carotenoid, lipid, and starch) present in *C. sorokiniana*, which could facilitate genetic modification to improve its productivity and reduce the reliance on other model organisms such as *Chlamydomonas*, since their miRNA expression profiles are different.

## 2. Materials and Methods

### 2.1. Samples

*Chlorella sorokiniana* (NIES-2168) used in this study was obtained from the Marine Biotechnology Lab at the Faculty of Agriculture, Universiti Putra Malaysia, which originated from the National Institute for Environmental Studies (NIES), Japan. The microalgae were cultured in Bold’s Basal Medium (BBM). At the beginning of microalga preculture preparation, the freshly cultured microalgae were first inoculated with an exponentially growing algal culture at 10% (*v*/*v*) and continuously illuminated at an intensity of approximately 10 µmol photons·m^−2^·s^−1^ with a shaking speed of 30 rpm at 27 °C. The preculture microalgae were allowed to grow until the mid-logarithmic phase, reaching approximately 2.5–3.0 × 10^6^ cells/mL on day 15. Then, microalgal cultures were further divided into two flasks with each flask containing 100 mL of 2 × 10^6^ cells/mL. One flask of cells was then cultured under normal conditions while the other was cultured under stress conditions.

The normal conditions for subsequent culturing were the same as the preculture conditions, whereas the stress culture conditions consisted of higher light intensity than the normal conditions (100 µmol photons·m^−2^·s^−1^) and the addition of 2% glucose [1,14]. Microalgae cultured under both conditions were allowed to grow for 7 days. The experiments were conducted in a shaker incubator, and a conical flask was used as the growth chamber; the white fluorescence light source was located above the cultures. All experiments were repeated independently in triplicate, generating six datasets for next-generation sequencing (NGS) analysis. The microalgae were harvested by separating the pellet from the medium via centrifugation at 8000× *g* for 10 min. The pellet was then flash-frozen using liquid nitrogen and stored at −20 °C until analysis.

### 2.2. Sample Preparation and Sequencing

Total RNA was extracted using Trizol^®^ (Invitrogen, Waltham, MA, USA) followed by clean-up using the modified RNeasy^®^Mini Kit (Qiagen, Hilden, Germany) [2]. Briefly, 100 mg of frozen tissue was ground using a prechilled mortar and pestle into a fine powder. The ground tissue sample was mixed with 1 mL of TRIzol reagent and homogenized until no visible debris remained. The homogenized samples were then incubated at room temperature for 5 min. Chloroform (0.2 mL) was added to the homogenate, vortexed vigorously for about 15 s, and incubated at room temperature for 2 to 3 min. After centrifugation, the aqueous layer of the sample was transferred to an RNeasy column from a mini RNA isolation kit (Qiagen, Hilden, Germany) for purification. Residual DNA was eliminated by performing on-column DNase I (Qiagen, Hilden, Germany) digestion at 37 °C for 30 min. The integrity of the extracted RNA was determined by gel electrophoresis, and its concentration was measured using a biospectrometer (Eppendorf BioSpectrometer, Hamburg, Germany).

A Qubit RNA HS (High-Sensitivity) assay kit (Life Technologies, Darmstadt, Germany) was used to determine the concentration of RNA in samples. Briefly, the RNA samples were diluted to a final concentration between 250 pg/µL and 100 ng/µL using the buffer provided before loading onto the Qubit 2.0 Fluorometer (Life Technologies, Darmstadt, Germany). The extracted RNA was analyzed using the Agilent High-Sensitivity RNA assay kit on the 2100 bioanalyzer (Agilent Technologies, Waldbronn, Germany) in complement with the RNA 6000 Nano LabChip kit. The assays were conducted for all normal and stress samples with three biological replicates. Data analysis was performed in accordance with the Agilent protocol.

### 2.3. Library Preparation

The library for small RNA-sequencing was prepared according to the NEBNext Multiplex Small RNA Library Prep Set for Illumina manual from New England Biolabs (NEB, Herts, UK). The main steps for the preparation of small RNA libraries using this kit were as follows: (1) 3′ ligation, (2) primer hybridization, (3) 5′ ligation, (4) first-strand synthesis, (5) PCR amplification, and (6) size selection. The molarity and size of the libraries were assessed by an Agilent high-sensitivity chip on a 2100 Bioanalyzer (Agilent Technologies, Germany). An individual library was prepared for each sample. Sequencing of 51 bp single-end reads was performed on the Illumina MiSeq at the Laboratory of Immunotherapeutic and Vaccine, Universiti Putra Malaysia.

### 2.4. Data Analysis

Raw reads obtained from Illumina MiSeq were first examined using FastQC. Reads containing adapter contaminants were removed by the data trimming program in the CLC Genomic Workbench Version 7.5.1 [15]. Only clean reads with a length between 18 nucleotides and 27 nucleotides were selected for further analysis. The clean reads from each *C. sorokiniana* library were annotated according to the miRBase version 22.1 and noncoding RNA (ncRNA) of *Chlamydomonas reinhardtii*, *Coccomyxa subellipsoidea* C-169, *Volvox carteri*, and *Ostreococcus lucimarinus*.

### 2.5. Quantitative PCR (qPCR) Validation

miR156c, miR164a, miR396c, and miR5645d that were identified in sequencing and significantly dysregulated in the *C. sorokiniana* cultured in stress conditions were subjected to quantitative PCR (qPCR) validation. Primers for the miR156c, miR164a, miR396c, and miR5645d were designed using miRprimer (Table S1) [16]. The primer for α-tubulin was designed using Primer-BLAST and used as the housekeeping gene for normalization. All primers were purchased from Integrated DNA Technologies (Singapore). Three replicates of total RNA extracted from *C. sorokiniana* cultured under normal and stress conditions were subjected to polyA tailing and reverse transcription using the miScript II RT kit. Briefly, 10 ng of total RNA was added with 5× miScript HiSpec buffer, 10× miScript nucleics mix, miScript reverse-transcriptase mix, and RNase-free water. The final mixture was incubated for 60 min at 37 °C, followed by inactivation of the miScript reverse-transcriptase mix via incubation for 5 min at 95 °C. For housekeeping gene amplification, 1 µg of total RNA was added with Quantiscript Reverse Transcriptase, 5× Quantiscript RT buffer, and RT primer mix. The final mixture was incubated for 15 min at 42 °C, followed by inactivation of Quantiscript reverse transcriptase via incubation for 3 min at 95 °C. The complementary DNA (cDNA) products synthesized using the miScript II RT kit and Quantitect RT kit were then diluted with 90 µL of RNase-free water. Subsequently, the cDNA synthesized from miRNA and mRNA was subjected to a qPCR run using the QuantiNova SYBR Green PCR kit (Qiagen, Venlo, The Netherlands) according to the manufacturer’s protocol [16]. Briefly, cDNA converted from three replicates of the sample was added with 1× QuantiNova SYBR Green PCR master mix (Qiagen, Venlo, The Netherlands), and 0.25 µM of each forward and reverse primer. The final mixture was immediately run using a BioRad CFX96 Real-Time PCR system (BioRad, Hercules, CA, USA) using the set-up of 95 °C for 2 min followed by 40 cycles of 95 °C for 5 s and 60 °C for 10 s. The melting curve was determined using a protocol of 95 °C for 1 min, 55 °C for 1 min, and 55 °C for 10 s at an increment of 0.5 °C per cycle for 80 cycles. The efficiency of the primers for miRNAs and housekeeping genes was tested by qPCR using cDNA synthesized from the stress samples, which were diluted to fivefold serial dilutions (5^1^, 5^2^, 5^3^, 5^4^, 5^5^) using the above qPCR set-up. A no-template control (NTC) and a no--reverse-transcription control (RTC) were included in each run of samples and standard curves. The primer efficiency was calculated using the standard curves, and the results are listed in Table S1. Differential expression (fold changes) of the miRNA between the normal and stress samples was normalized using α-tubulin as a housekeeping gene with the double delta Ct method, and the results were compared between sequencing and qPCR analyses. Statistical analysis was performed using SPSS version 17.0 (IBM Corp, Armonkk, NY, USA). The comparison between normal and stress samples was investigated using Student’s *t*-tests. A value of *p* < 0.05 was considered statistically significant.

### 2.6. Prediction of miRNA Targets and Functional Analysis

Predicted miRNAs were identified using psRNATarget: A Plant Small RNA Target Analysis Server [17] using the *Chlamydomonas reinhardtii* JGI genome project Phytozome (phytozome v10.0, internal number 281) [18]. Targets were qualified on the basis of the default scoring schema with the following criteria: (a) expectation scores between 0 and 5, (b) unpaired energy score (UPE) ranging from 0.0 to 25.0, (c) high-scoring segment pair (HSP) size of 19 nucleotides, (d) maximum number of transcripts targeted less than or equal to 200 hits, (e) seed region ranging from two to 13 nucleotides, and (f) central mismatch leading to translational inhibition centered on two nucleotides. The information pertaining to transcripts targeted by the miRNA includes the type of inhibition (cleavage or translation repression) and multiplicity (number of sites in the transcript likely to be targeted by the miRNA).

Functional analysis of miRNA targets was carried out using AlgaePath [19]. The query input obtained from psRNATarget results was submitted to the gene group analysis. Information on protein class and the pathways related to miRNA targeting genes were also generated using the same tool.

## 3. Results

### 3.1. High-Throughput Small RNA-Sequencing Analysis

**Figure 1:**
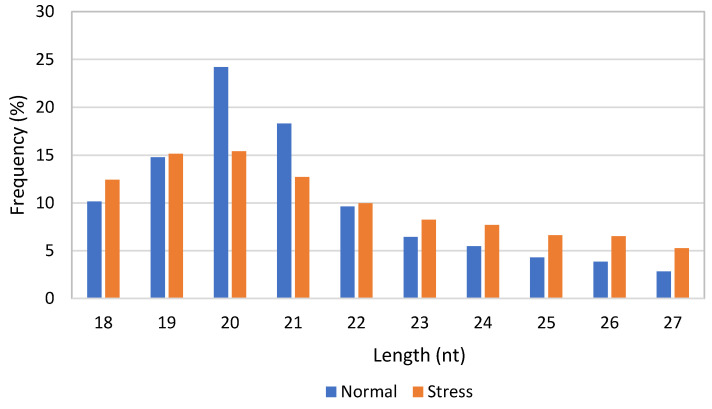
Length distribution of small RNAs in normal and stress-induced *C. sorokiniana* libraries.

Small RNA-sequencing for the normal and stress-induced *C. sorokiniana* libraries generated approximately 2,440,339 and 3,597,169 reads, respectively. The numbers of cleans reads, annotated samples, and unannotated samples are shown in Table 1. Approximately 96,553 and 248,139 unique reads were produced from normal and stress libraries, respectively. The length distribution of the small RNAs in the normal and stress libraries is shown in Figure 1. The size distribution indicated that the most frequent size length was 20 nt, followed by 21 nt and 19 nt, in both libraries.

### 3.2. Identification of miRNAs in *C. sorokiniana*

**Figure 2:**
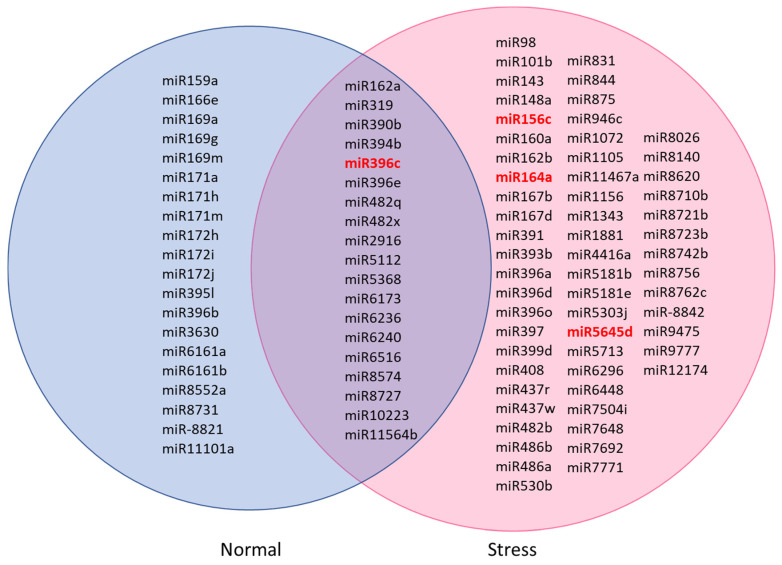
Common and specific miRNAs identified in normal and stress-induced *C. sorokiniana* libraries. Conserved miRNAs were miR156 to miR408 [20]. Others were nonconserved miRNAs. The red miRNAs were selected for quantitative PCR (qPCR) validation.

A Venn diagram was generated to show the number of miRNAs found in normal and stress-induced *C. sorokiniana* libraries (Figure 2). From 98 miRNAs annotated in the database, 20 miRNAs were identified in the normal *C. sorokiniana* library, while 59 miRNAs were found in the stress-induced *C. sorokiniana* library. A total of 19 miRNAs were found to be common in both libraries.

### 3.3. qPCR Validation of the Selected miRNAs between Stress-Induced and Normal *C. sorokiniana*

**Figure 3:**
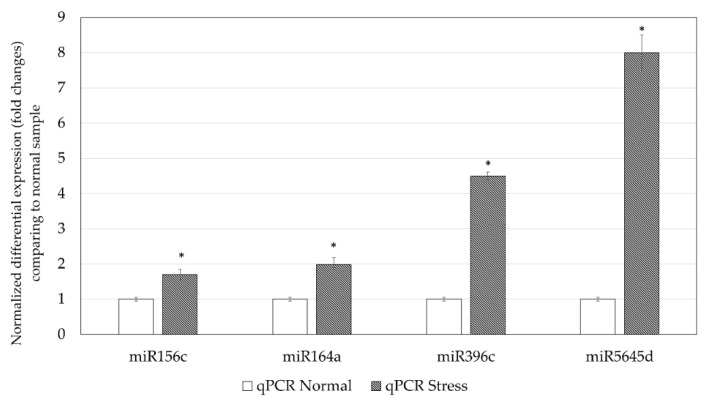
Normalized fold changes of miR156c, miR164a, miR396c and miR5645d were quantified using qPCR. * *p* < 0.05 when compared with the normal sample.

The expressions of miR156c, miR164a, miR396c, and miR5645d of *C. sorokiniana* cultured under normal and stress conditions were validated by qPCR. Figure 2 shows that miR156c, miR164a, and miR5645d were only detected in the *C. sorokiniana* cultured under stress conditions. miR396c was detected in samples cultured under both conditions and was fourfold higher in the stress sample. qPCR was able to detect all miRNAs in both normal and stress samples; the expression of these selected microRNAs was upregulated under stress conditions, indicating that these miRNAs were overexpressed under stress conditions (Figure 3).

### 3.4. Functional Analysis of the Putative Target Genes of miRNAs in *C. sorokiniana*

**Figure 4:**
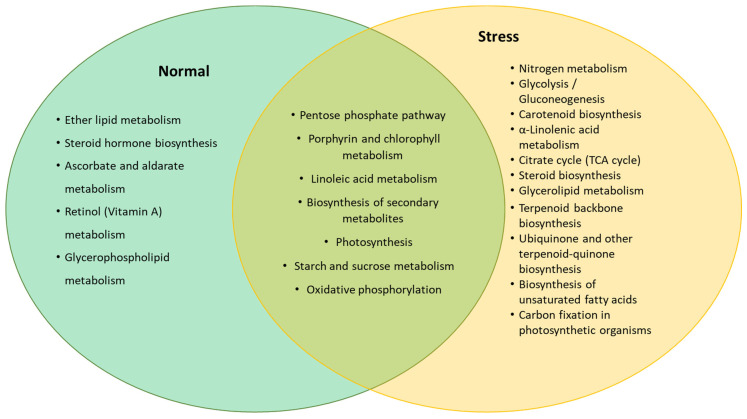
Predicted pathways enriched by genes targeted by the miRNA expressed in normal and stress conditions.

A total of 4813 miRNA target genes were predicted and identified for known (or conserved) miRNAs (Supplementary Data 1) using psRNAtarget tools. These target genes were then subjected to functional annotation using AlgaePath, and the results obtained are presented in Table 2. From the results obtained, 14 pathways were identified as significantly enriched (*p* < 0.05). Functional analysis of the miRNA transcriptome of *C. sorokiniana* demonstrated that the highest percentage of the miRNA target genes found with significant functional annotation were involved in the biosynthesis of secondary metabolites. Additionally, the second highest percentage of the target genes were involved in purine metabolism, followed by cell cycle, porphyrin and chlorophyll metabolism, aminoacyl-transfer RNA (tRNA) biosynthesis, RNA degradation, and the pentose phosphate pathway. The other important metabolism involved in the regulation of miRNA identified in *C. sorokiniana* miRNA transcriptome were ether lipid metabolism, starch and sucrose metabolism, carotenoid biosynthesis, glycerolipid metabolism, isoprenoid metabolism (ubiquitin and terpenoid biosynthesis), and others (Table S2). Pathways enriched in different cultivation conditions (normal and stress) are presented in Figure 4. From the figure, more pathways related to the production of secondary metabolites were enriched in stress-induced samples, such as carotenoid biosynthesis, terpenoid backbone biosynthesis, and ubiquinone and other terpenoid–quinone biosynthesis. Meanwhile, different pathways involved in fat and lipid syntheses were observed in *C. sorokiniana* cultured in normal and stress conditions. For example, ether lipid and glycerophospholipid metabolism were enriched in normal conditions, while α-linolenic acid metabolism, glycerolipid metabolism, and biosynthesis of unsaturated fatty acid pathways were enriched in the stress sample (Table S3).

## 4. Discussion

### 4.1. Characteristics of miRNA in Microalgae

Based on Qin et al. [20], most of the well-studied and functionally known miRNAs are conserved and designated with identification numbers ranging from miR156 to miR408. The identified miRNAs are commonly found in a variety of terrestrial plants and nonflowering moss. The miRNAs that are species-specific were identified as nonconserved miRNAs. Nonconserved miRNAs are usually imprecisely processed and weakly expressed, and they lack known functional targets. However, some of the nonconserved miRNAs may exhibit higher expression levels under particular conditions, such as environmental changes [20,21]. However, there is controversy regarding the homology of miRNAs in terrestrial plants and algae [22,23]. A recent review by De [24] indicated that miRNAs involved in key biotic signaling networks of streptophyte algae and terrestrial algae might have similar functions. Furthermore, there were reports on the similarity of target function in green alga *Chlamydomonas* and animals [16]. Previous findings demonstrated that miRNAs found in animals, plants, and algae share similar biogenesis, function, and turnover pathways in diverse lineages [16,25,26].

The average length of detected miRNA in this study (20 nt) is almost similar to the length of plant miRNA. Generally, the length of miRNA is between 16 and 24 nt, whereby plant miRNA is around 21 nt in length and animal miRNA is a little longer at 22–24 nt. The range of selected miRNA length in this study was based on the range of plant and animal miRNA [27,28]. Previous studies reported that more than 60% of all plant miRNAs are 21 nt in length [8]. After the miRNA identification step, the subsequent step was to infer the roles of the putative target genes, as well as the regulatory networks they are involved in. Basically, miRNA binds to the target mRNA and regulates gene expression by cleaving or inhibiting the translation of the target mRNA. In this study, conserved miRNAs were predicted on the basis of a comparison with the reference library. From the result obtained through identification and functional analysis of target miRNAs, it was revealed that most of the identified miRNAs in both normal and stress-induced libraries of this study were significantly involved in the regulation of secondary metabolite biosynthesis.

### 4.2. Functional Analysis of Identified miRNA in *C. sorokiniana*

Several identified miRNAs in this study might be involved in regulating flavonoid biosynthesis, terpenoid biosynthesis, and alkaloid biosynthesis [29]. For instance, miR156 in *Arabidopsis thaliana* was shown to regulate metabolic flux during the flavonoid biosynthetic pathway by targeting the SQUAMOSA promoter-binding protein-like (SPL) protein. According to previous findings, a higher concentration of miR156 reduced SPL activity and enhanced the anthocyanin biosynthetic genes [30]. Wei et al. [31] demonstrated that the expression of *Arabidopsis* miR156 in *Brassica napus* caused an increased level of carotenoid production and reproductive shoot branching, suggesting a link between miR156 expression and carotenoid metabolism. The ability of microalgae to produce certain carotenoids can be explored by investigating miR156 expression under stress-related conditions. In addition, miR172i was identified as targeting mRNAs coding for enzymes of the phenylpropanoid pathway, such as 4-coumarate–CoA ligase, chalcone synthase, caffeoyl-CoA *O*-methyl transferase, dihydroflavonol 4-reductase, and C,2-hydroxyisoflavanone dehydratase in *Podophyllum hexandrum* [32]. miR408 identified in the stress-induced library is involved in regulating the alkaloid biosynthetic pathway in the opium poppy, which targets the mRNA of reticuline oxidase-like protein that converts *S*-reticuline to (*S*)-scoulerine in benzylisoquinoline alkaloid biosynthesis [33].

It is worth noting that the identification of miR164 in the stress-induced library from this study might be related to the disruption of chlorophyll content. In a previous report, miR164 was found to regulate chlorophyll breakdown during leaf senescence in *Arabidopsis thaliana* [34,35] by targeting the no apical meristem, ATAF ½ and cup-shaped cotyledon (NAC) transcription factor involved in chlorophyll catabolism. Similarly, in this study, the chlorophyll content of *C. sorokiniana* grown under stress-induced conditions was reduced by 80% compared to cultures grown under normal conditions [3]. These findings demonstrate a possible correlation between the predicted function of the identified miRNA and the morphological study.

Other interesting miRNAs found in this study were predicted to have similar functions to miRNAs involved in regulating lipid biosynthesis in higher organisms, such as animals and plants. miR148a identified from the stress-induced library, which is highly expressed in hepatic tissue of mouse and human, is responsible for alteration of total cholesterol (TC), low-density-lipoprotein-cholesterol (LDL-C), and triglycerides (TAGs) in blood [36]. It was reported that miR148a is involved in regulating the expression of carnitine palmitoyltransferase 1 (CPT1A), a central regulator of mitochondrial fatty-acid β-oxidation [37]. Meanwhile, in oil-producing plants such as oil palm and rapeseed, miR156, miR159, miR166, miR167, miR172, and miR397 were identified in regulating lipid metabolism [38,39]. miR156 and miR397 in oil palm plants are known to regulate fatty-acid synthesis in plastids and the transport of fatty acyl-ACP from plastids to the endoplasmic reticulum by targeting acetyl-CoA carboxylase 1 (ACC1), long-chain acyl-CoA synthetase 9 (LACS9), LACS4, and enoyl-ACP reductase (ENR). In rapeseed plants, miR156 and miR167 are involved in the regulation of oil content during seed development and maturation by targeting SPL and the adenosine diphosphate ribosylation factor (ARF) family. miR172 from rapeseed plants targets the zinc finger protein associated with fatty-acid synthesis and metabolism. The other miRNAs, such as miR396 identified in the stress-induced library, were reported to target a conserved domain of *GRF* and the WRC (Trp, Arg, Cys) domain, which contains a functional nuclear localization signal and a putative zinc finger motif [40]. This miRNA plays regulatory roles in plant growth and development, as well as the responses to various environmental stresses [41,42]. This is in agreement with the current study, in which higher numbers of miR396 were identified in the stress-induced library, namely, miR396a, miR396c, miR396d, and miR396o, as compared to only miR396b identified in the normal library.

An understanding of the mechanism of reaction in microalgae to various environmental factors and of the information on RNA-mediated stress regulatory networks offers an innovative path for enhancing the production of metabolites in *C. sorokiniana*. Suitable stress conditions could stimulate the synthesis and accumulation of lipids and high-value products derived from this microalga. The considerable amount of available omics data from green microalgae could be channeled toward achieving efficient regulation of metabolism via the application of synthetic biology by targeting the regulatory networks rather than deletion or overexpression of enzyme-coding genes [43,44].

## 5. Conclusions

The present report is the first to identify and profile the expression pattern of miRNA in *C. sorokiniana* through high-throughput sequencing. A total of 98 miRNAs were identified in the small RNA transcriptome of *C. sorokiniana* grown under normal and stress conditions. Functional analysis of target miRNAs genes demonstrated that most of the identified miRNAs are involved in the regulation of secondary metabolite biosynthesis. The production of secondary metabolites, such as carotenoid and terpenoid biosynthesis, as well as lipid production, was mostly found in stress samples. These findings provide new insights into the molecular mechanisms involved in the regulation of metabolite production toward stress stimulus in microalgae.

## Figures and Tables

**Table 1:** Statistic for deep-sequencing results generated from normal and stress-induced *Chlorella sorokiniana* libraries. miRNA: microRNA.

		Total Reads	Percentage	Unique Reads	Percentage
**Normal**	**Raw reads**	2,440,339			
	**Clean reads**	1,004,551	100	96,553	100
	**miRNA**	3191	0.28	422	0.44
	**Others**	232	0.02	59	0.06
	**Unannotated**	1,001,128	99.7	96,072	99.5
**Stress**	**Raw reads**	3,597,169			
	**Clean reads**	1,511,785	100	248,139	100
	**miRNA**	6688	0.44	788	0.32
	**Others**	615	0.041	215	0.08
	**Unannotated**	1,504,482	99.52	247,136	99.6

**Table 2:** Functional analysis using Algaepath of known miRNA targets in *C. sorokiniana*. Results show only significant functional annotation with *p* < 0.05. tRNA: transfer RNA.

Pathway ID	Pathway Name	Hit Number (Query)	Percentage in Query	*p*-Value
map01110	Biosynthesis of secondary metabolites	43	4.08%	0.0015
map00230	Purine metabolism	17	1.61%	0.0324
map04110	Cell cycle	10	0.95%	0.0077
map00860	Porphyrin and chlorophyll metabolism	9	0.85%	0.0018
map00970	Aminoacyl-tRNA biosynthesis	8	0.76%	0.0282
map03018	RNA degradation	8	0.76%	0.0406
map00030	Pentose phosphate pathway	7	0.66%	0.0041
map03050	Proteasome	7	0.66%	0.0315
map04712	Circadian rhythm, plant	5	0.47%	0.0004
map00450	Selenocompound metabolism	5	0.47%	0.0092
map00982	Drug metabolism, cytochrome P450	5	0.47%	0.03
map00983	Drug metabolism, other enzymes	5	0.47%	0.0362
map00290	Valine, leucine, and isoleucine biosynthesis	4	0.38%	0.0155
map00770	Pantothenate and CoA biosynthesis	4	0.38%	0.0531
map00565	Ether lipid metabolism	3	0.28%	0.0056

## Supplementary Materials

The following are available online at https://www.mdpi.com/2073-4425/11/10/1131/s1; Supplementary Data 1: A total of 4813 miRNA target genes were predicted and identified for known (or conserved) miRNAs; Table S1: MicroRNA primer sequence for qPCR; Table S2: AlgaePath analysis; Table S3: AlgaePath analysis for Normal sample and Stress sample.
