# Supplementary material for: Identification and Analysis of microRNAs in Chlorella sorokiniana Using High-Throughput Sequencing
Source: Genes (Basel). 2020 Sep 25;11(10):1131. doi: 10.3390/genes11101131 (PMC7599482; doi:10.3390/genes11101131)
Supplement: Supplementary file 1 [file genes-11-01131-s001.zip › Supplementary Table S1.docx]

**Supplementary Table S1**

Primer sequences of microRNAs and housekeeping gene (HKG) for qPCR.

|  | **Forward (5’-3’)** | **Reverse (5’-3’)** | **qPCR Efficiency (%)** |
| --- | --- | --- | --- |
| **miR156c** | GGTTCAGCATGGACGAG | GGTCCAGTTTTTTTTTTTTTTTCAC | 95.1 |
| **miR164a** | AGCAAAGCGGCTGC | GGTCCAGTTTTTTTTTTTTTTTCAC | 92.3 |
| **miR396c** | CGCAGAAGCTGTGGGAT | AGGTCCAGTTTTTTTTTTTTTTTGAA | 91.6 |
| **miR5645d** | GCAGCGCAGGTCTTTTTTAAA | AGGTCCAGTTTTTTTTTTTTTTTCTT | 104.5 |
| **Alpha-tubulin (HKG)** | CACAGTTTACCCGTCTCCCC | TCAGCCGGTTAAGGTTGGTG | 93.4 |
