# Supplementary material for: Identification and Analysis of microRNAs in Chlorella sorokiniana Using High-Throughput Sequencing
Source: Genes (Basel). 2020 Sep 25;11(10):1131. doi: 10.3390/genes11101131 (PMC7599482; doi:10.3390/genes11101131)
Supplement: Supplementary file 1 [file genes-11-01131-s001.zip › Supplementary Table S2.docx]

**Supplementary Table S2** AlgaePath analysis.

| **Pathway ID** | **Pathway name** | **Hit number (Query)** | **Percentage in query** | **P-value** |
| --- | --- | --- | --- | --- |
| map01100 | Metabolic pathways | 73 | 6.93 % | 0.0621 |
| map01110 | Biosynthesis of secondary metabolites | 43 | 4.08 % | 0.0015 |
| map01120 | Microbial metabolism in diverse environments | 18 | 1.71 % | 0.2198 |
| map00230 | Purine metabolism | 17 | 1.61 % | 0.0324 |
| map03040 | Spliceosome | 12 | 1.14 % | 0.1145 |
| map04110 | Cell cycle | 10 | 0.95 % | 0.0077 |
| map00860 | Porphyrin and chlorophyll metabolism | 9 | 0.85 % | 0.0018 |
| map04111 | Cell cycle - yeast | 9 | 0.85 % | 0.0111 |
| map00240 | Pyrimidine metabolism | 9 | 0.85 % | 0.1762 |
| map00970 | Aminoacyl-tRNA biosynthesis | 8 | 0.76 % | 0.0282 |
| map03018 | RNA degradation | 8 | 0.76 % | 0.0406 |
| map00030 | Pentose phosphate pathway | 7 | 0.66 % | 0.0041 |
| map03050 | Proteasome | 7 | 0.66 % | 0.0315 |
| map04120 | Ubiquitin mediated proteolysis | 7 | 0.66 % | 0.1822 |
| map03010 | Ribosome | 7 | 0.66 % | 0.8449 |
| map00680 | Methane metabolism | 6 | 0.57 % | 0.1387 |
| map04113 | Meiosis - yeast | 6 | 0.57 % | 0.1509 |
| map03013 | RNA transport | 6 | 0.57 % | 0.6331 |
| map04712 | Circadian rhythm - plant | 5 | 0.47 % | 0.0004 |
| map00450 | Selenocompound metabolism | 5 | 0.47 % | 0.0092 |
| map00982 | Drug metabolism - cytochrome P450 | 5 | 0.47 % | 0.03 |
| map00983 | Drug metabolism - other enzymes | 5 | 0.47 % | 0.0362 |
| map00480 | Glutathione metabolism | 5 | 0.47 % | 0.0888 |
| map04142 | Lysosome | 5 | 0.47 % | 0.1121 |
| map04144 | Endocytosis | 5 | 0.47 % | 0.1248 |
| map01210 | 2-Oxocarboxylic acid metabolism | 5 | 0.47 % | 0.1381 |
| map00500 | Starch and sucrose metabolism | 5 | 0.47 % | 0.1381 |
| map03015 | mRNA surveillance pathway | 5 | 0.47 % | 0.3154 |
| map03008 | Ribosome biogenesis in eukaryotes | 5 | 0.47 % | 0.5437 |
| map00290 | Valine, leucine and isoleucine biosynthesis | 4 | 0.38 % | 0.0155 |
| map00770 | Pantothenate and CoA biosynthesis | 4 | 0.38 % | 0.0531 |
| map03022 | Basal transcription factors | 4 | 0.38 % | 0.1658 |
| map03060 | Protein export | 4 | 0.38 % | 0.2206 |
| map00270 | Cysteine and methionine metabolism | 4 | 0.38 % | 0.2596 |
| map04145 | Phagosome | 4 | 0.38 % | 0.2796 |
| map03030 | DNA replication | 4 | 0.38 % | 0.3202 |
| map04114 | Oocyte meiosis | 4 | 0.38 % | 0.3612 |
| map00260 | Glycine, serine and threonine metabolism | 4 | 0.38 % | 0.4021 |
| map00190 | Oxidative phosphorylation | 4 | 0.38 % | 0.8018 |
| map00565 | Ether lipid metabolism | 3 | 0.28 % | 0.0056 |
| map04270 | Vascular smooth muscle contraction | 3 | 0.28 % | 0.1348 |
| map00340 | Histidine metabolism | 3 | 0.28 % | 0.1348 |
| map02010 | ABC transporters | 3 | 0.28 % | 0.1348 |
| map00980 | Metabolism of xenobiotics by cytochrome P450 | 3 | 0.28 % | 0.2242 |
| map04721 | Synaptic vesicle cycle | 3 | 0.28 % | 0.2971 |
| map00350 | Tyrosine metabolism | 3 | 0.28 % | 0.3218 |
| map00564 | Glycerophospholipid metabolism | 3 | 0.28 % | 0.3218 |
| map00020 | Citrate cycle (TCA cycle) | 3 | 0.28 % | 0.4672 |
| map00330 | Arginine and proline metabolism | 3 | 0.28 % | 0.6721 |
| map00710 | Carbon fixation in photosynthetic organisms | 3 | 0.28 % | 0.6721 |
| map03420 | Nucleotide excision repair | 3 | 0.28 % | 0.6891 |
| map04141 | Protein processing in endoplasmic reticulum | 3 | 0.28 % | 0.9683 |
| map00624 | Polycyclic aromatic hydrocarbon degradation | 2 | 0.19 % | 0.089 |
| map04064 | NF-kappa B signaling pathway | 2 | 0.19 % | 0.089 |
| map00960 | Tropane, piperidine and pyridine alkaloid biosynthesis | 2 | 0.19 % | 0.1176 |
| map04340 | Hedgehog signaling pathway | 2 | 0.19 % | 0.1176 |
| map03070 | Bacterial secretion system | 2 | 0.19 % | 0.1482 |
| map04390 | Hippo signaling pathway | 2 | 0.19 % | 0.1482 |
| map00591 | Linoleic acid metabolism | 2 | 0.19 % | 0.213 |
| map04961 | Endocrine and other factor-regulated calcium reabsorption | 2 | 0.19 % | 0.213 |
| map04210 | Apoptosis | 2 | 0.19 % | 0.213 |
| map00906 | Carotenoid biosynthesis | 2 | 0.19 % | 0.2463 |
| map00410 | beta-Alanine metabolism | 2 | 0.19 % | 0.2798 |
| map04115 | p53 signaling pathway | 2 | 0.19 % | 0.3132 |
| map00052 | Galactose metabolism | 2 | 0.19 % | 0.3463 |
| map00130 | Ubiquinone and other terpenoid-quinone biosynthesis | 2 | 0.19 % | 0.3788 |
| map04130 | SNARE interactions in vesicular transport | 2 | 0.19 % | 0.4106 |
| map00561 | Glycerolipid metabolism | 2 | 0.19 % | 0.5009 |
| map00400 | Phenylalanine, tyrosine and tryptophan biosynthesis | 2 | 0.19 % | 0.529 |
| map04310 | Wnt signaling pathway | 2 | 0.19 % | 0.529 |
| map00910 | Nitrogen metabolism | 2 | 0.19 % | 0.556 |
| map00900 | Terpenoid backbone biosynthesis | 2 | 0.19 % | 0.556 |
| map00051 | Fructose and mannose metabolism | 2 | 0.19 % | 0.556 |
| map00280 | Valine, leucine and isoleucine degradation | 2 | 0.19 % | 0.556 |
| map00630 | Glyoxylate and dicarboxylate metabolism | 2 | 0.19 % | 0.5819 |
| map03020 | RNA polymerase | 2 | 0.19 % | 0.5819 |
| map03430 | Mismatch repair | 2 | 0.19 % | 0.6303 |
| map00250 | Alanine, aspartate and glutamate metabolism | 2 | 0.19 % | 0.6947 |
| map00195 | Photosynthesis | 2 | 0.19 % | 0.6947 |
| map04146 | Peroxisome | 2 | 0.19 % | 0.8924 |
| map00010 | Glycolysis / Gluconeogenesis | 2 | 0.19 % | 0.92 |
| map04711 | Circadian rhythm - fly | 1 | 0.09 % | 0.091 |
| map01051 | Biosynthesis of ansamycins | 1 | 0.09 % | 0.091 |
| map04514 | Cell adhesion molecules (CAMs) | 1 | 0.09 % | 0.1663 |
| map00232 | Caffeine metabolism | 1 | 0.09 % | 0.1663 |
| map00660 | C5-Branched dibasic acid metabolism | 1 | 0.09 % | 0.3047 |
| map00511 | Other glycan degradation | 1 | 0.09 % | 0.3651 |
| map04360 | Axon guidance | 1 | 0.09 % | 0.3651 |
| map04660 | T cell receptor signaling pathway | 1 | 0.09 % | 0.3651 |
| map04662 | B cell receptor signaling pathway | 1 | 0.09 % | 0.3651 |
| map04975 | Fat digestion and absorption | 1 | 0.09 % | 0.4204 |
| map04012 | ErbB signaling pathway | 1 | 0.09 % | 0.4204 |
| map04062 | Chemokine signaling pathway | 1 | 0.09 % | 0.4204 |
| map04391 | Hippo signaling pathway - fly | 1 | 0.09 % | 0.4204 |
| map00950 | Isoquinoline alkaloid biosynthesis | 1 | 0.09 % | 0.4708 |
| map04722 | Neurotrophin signaling pathway | 1 | 0.09 % | 0.4708 |
| map00750 | Vitamin B6 metabolism | 1 | 0.09 % | 0.4708 |
| map04962 | Vasopressin-regulated water reabsorption | 1 | 0.09 % | 0.5169 |
| map04976 | Bile secretion | 1 | 0.09 % | 0.5169 |
| map04330 | Notch signaling pathway | 1 | 0.09 % | 0.5169 |
| map00310 | Lysine degradation | 1 | 0.09 % | 0.5169 |
| map04666 | Fc gamma R-mediated phagocytosis | 1 | 0.09 % | 0.559 |
| map00563 | Glycosylphosphatidylinositol(GPI)-anchor biosynthesis | 1 | 0.09 % | 0.559 |
| map04510 | Focal adhesion | 1 | 0.09 % | 0.559 |
| map04916 | Melanogenesis | 1 | 0.09 % | 0.559 |
| map04725 | Cholinergic synapse | 1 | 0.09 % | 0.559 |
| map00140 | Steroid hormone biosynthesis | 1 | 0.09 % | 0.5975 |
| map00592 | alpha-Linolenic acid metabolism | 1 | 0.09 % | 0.6326 |
| map00360 | Phenylalanine metabolism | 1 | 0.09 % | 0.6326 |
| map00627 | Aminobenzoate degradation | 1 | 0.09 % | 0.6326 |
| map00100 | Steroid biosynthesis | 1 | 0.09 % | 0.6647 |
| map04623 | Cytosolic DNA-sensing pathway | 1 | 0.09 % | 0.6647 |
| map01040 | Biosynthesis of unsaturated fatty acids | 1 | 0.09 % | 0.7451 |
| map04728 | Dopaminergic synapse | 1 | 0.09 % | 0.7451 |
| map00053 | Ascorbate and aldarate metabolism | 1 | 0.09 % | 0.7451 |
| map00830 | Retinol metabolism | 1 | 0.09 % | 0.7674 |
| map00720 | Carbon fixation pathways in prokaryotes | 1 | 0.09 % | 0.7878 |
| map04910 | Insulin signaling pathway | 1 | 0.09 % | 0.8063 |
| map04626 | Plant-pathogen interaction | 1 | 0.09 % | 0.8233 |
| map00562 | Inositol phosphate metabolism | 1 | 0.09 % | 0.8388 |
| map03440 | Homologous recombination | 1 | 0.09 % | 0.8529 |
| map04151 | PI3K-Akt signaling pathway | 1 | 0.09 % | 0.8658 |
| map04914 | Progesterone-mediated oocyte maturation | 1 | 0.09 % | 0.8776 |
| map03460 | Fanconi anemia pathway | 1 | 0.09 % | 0.8982 |
