# Supplementary material for: Identification and Analysis of microRNAs in Chlorella sorokiniana Using High-Throughput Sequencing
Source: Genes (Basel). 2020 Sep 25;11(10):1131. doi: 10.3390/genes11101131 (PMC7599482; doi:10.3390/genes11101131)
Supplement: Supplementary file 1 [file genes-11-01131-s001.zip › Supplementary Table S3.docx]

**Supplementary Table S3** AlgaePath analysis for Normal sample and Stress sample.

**Normal:**

| **Pathway ID** | **Pathway name** | **Hit number (Query)** | **Percentage in query** | **P-value** |
| --- | --- | --- | --- | --- |
| map00480 | Glutathione metabolism | 3 | 1.46% | 0.0103 |
| map00232 | Caffeine metabolism | 1 | 0.49% | 0.0329 |
| map00980 | Metabolism of xenobiotics by cytochrome P450 | 2 | 0.98% | 0.0383 |
| map03018 | RNA degradation | 3 | 1.46% | 0.0389 |
| map00982 | Drug metabolism - cytochrome P450 | 2 | 0.98% | 0.0461 |
| map00983 | Drug metabolism - other enzymes | 2 | 0.98% | 0.0502 |
| map00030 | Pentose phosphate pathway | 2 | 0.98% | 0.0632 |
| map03040 | Spliceosome | 4 | 1.95% | 0.069 |
| map00565 | Ether lipid metabolism | 1 | 0.49% | 0.0804 |
| map00860 | Porphyrin and chlorophyll metabolism | 2 | 0.98% | 0.1078 |
| map00960 | Tropane, piperidine and pyridine alkaloid biosynthesis | 1 | 0.49% | 0.1108 |
| map03050 | Proteasome | 2 | 0.98% | 0.1187 |
| map00260 | Glycine, serine and threonine metabolism | 2 | 0.98% | 0.1242 |
| map00140 | Steroid hormone biosynthesis | 1 | 0.49% | 0.1545 |
| map00591 | Linoleic acid metabolism | 1 | 0.49% | 0.1545 |
| map00970 | Aminoacyl-tRNA biosynthesis | 2 | 0.98% | 0.1586 |
| map00627 | Aminobenzoate degradation | 1 | 0.49% | 0.1686 |
| map00290 | Valine, leucine and isoleucine biosynthesis | 1 | 0.49% | 0.1825 |
| map00410 | beta-Alanine metabolism | 1 | 0.49% | 0.1825 |
| map01100 | Metabolic pathways | 15 | 7.32% | 0.1879 |
| map00053 | Ascorbate and aldarate metabolism | 1 | 0.49% | 0.2228 |
| map00830 | Retinol metabolism | 1 | 0.49% | 0.2358 |
| map04130 | SNARE interactions in vesicular transport | 1 | 0.49% | 0.2358 |
| map00770 | Pantothenate and CoA biosynthesis | 1 | 0.49% | 0.2486 |
| map00562 | Inositol phosphate metabolism | 1 | 0.49% | 0.2858 |
| map01110 | Biosynthesis of secondary metabolites | 7 | 3.41% | 0.2867 |
| map04721 | Synaptic vesicle cycle | 1 | 0.49% | 0.3096 |
| map01120 | Microbial metabolism in diverse environments | 4 | 1.95% | 0.3144 |
| map00564 | Glycerophospholipid metabolism | 1 | 0.49% | 0.3212 |
| map00230 | Purine metabolism | 3 | 1.46% | 0.335 |
| map03022 | Basal transcription factors | 1 | 0.49% | 0.3438 |
| map03013 | RNA transport | 2 | 0.98% | 0.3499 |
| map00195 | Photosynthesis | 1 | 0.49% | 0.3657 |
| map04142 | Lysosome | 1 | 0.49% | 0.3973 |
| map04145 | Phagosome | 1 | 0.49% | 0.4074 |
| map00500 | Starch and sucrose metabolism | 1 | 0.49% | 0.4174 |
| map03420 | Nucleotide excision repair | 1 | 0.49% | 0.4918 |
| map04146 | Peroxisome | 1 | 0.49% | 0.5089 |
| map04120 | Ubiquitin mediated proteolysis | 1 | 0.49% | 0.6004 |
| map00190 | Oxidative phosphorylation | 1 | 0.49% | 0.652 |
| map00240 | Pyrimidine metabolism | 1 | 0.49% | 0.7125 |

**Stress:**

| **Pathway ID** | **Pathway name** | **Hit number (Query)** | **Percentage in query** | **P-value** |
| --- | --- | --- | --- | --- |
| map04712 | Circadian rhythm - plant | 4 | 0.60% | 0.0009 |
| map00450 | Selenocompound metabolism | 5 | 0.75% | 0.0012 |
| map00680 | Methane metabolism | 6 | 0.89% | 0.0226 |
| map00290 | Valine, leucine and isoleucine biosynthesis | 3 | 0.45% | 0.0249 |
| map01210 | 2-Oxocarboxylic acid metabolism | 5 | 0.75% | 0.0284 |
| map00030 | Pentose phosphate pathway | 4 | 0.60% | 0.0452 |
| map02010 | ABC transporters | 3 | 0.45% | 0.0457 |
| map04270 | Vascular smooth muscle contraction | 3 | 0.45% | 0.0457 |
| map04110 | Cell cycle | 6 | 0.89% | 0.0494 |
| map03070 | Bacterial secretion system | 2 | 0.30% | 0.0679 |
| map00270 | Cysteine and methionine metabolism | 4 | 0.60% | 0.0794 |
| map04142 | Lysosome | 4 | 0.60% | 0.0794 |
| map00970 | Aminoacyl-tRNA biosynthesis | 5 | 0.75% | 0.0843 |
| map03015 | mRNA surveillance pathway | 5 | 0.75% | 0.0843 |
| map00500 | Starch and sucrose metabolism | 4 | 0.60% | 0.0959 |
| map04961 | Endocrine and other factor-regulated calcium reabsorption | 2 | 0.30% | 0.1016 |
| map00982 | Drug metabolism - cytochrome P450 | 3 | 0.45% | 0.1054 |
| map04514 | Cell adhesion molecules (CAMs) | 1 | 0.15% | 0.1074 |
| map01110 | Biosynthesis of secondary metabolites | 23 | 3.43% | 0.1128 |
| map00230 | Purine metabolism | 10 | 1.49% | 0.135 |
| map03022 | Basal transcription factors | 3 | 0.45% | 0.156 |
| map04120 | Ubiquitin mediated proteolysis | 5 | 0.75% | 0.1736 |
| map00052 | Galactose metabolism | 2 | 0.30% | 0.1785 |
| map03008 | Ribosome biogenesis in eukaryotes | 5 | 0.75% | 0.1928 |
| map00340 | Histidine metabolism | 2 | 0.30% | 0.1989 |
| map00660 | C5-Branched dibasic acid metabolism | 1 | 0.15% | 0.2032 |
| map04111 | Cell cycle - yeast | 4 | 0.60% | 0.2219 |
| map00770 | Pantothenate and CoA biosynthesis | 2 | 0.30% | 0.2404 |
| map03018 | RNA degradation | 4 | 0.60% | 0.2462 |
| map03030 | DNA replication | 3 | 0.45% | 0.2728 |
| map04975 | Fat digestion and absorption | 1 | 0.15% | 0.2889 |
| map04064 | NF-kappa B signaling pathway | 1 | 0.15% | 0.2889 |
| map00624 | Polycyclic aromatic hydrocarbon degradation | 1 | 0.15% | 0.2889 |
| map00400 | Phenylalanine, tyrosine and tryptophan biosynthesis | 2 | 0.30% | 0.3033 |
| map04114 | Oocyte meiosis | 3 | 0.45% | 0.3037 |
| map00051 | Fructose and mannose metabolism | 2 | 0.30% | 0.3241 |
| map00910 | Nitrogen metabolism | 2 | 0.30% | 0.3241 |
| map00280 | Valine, leucine and isoleucine degradation | 2 | 0.30% | 0.3241 |
| map04340 | Hedgehog signaling pathway | 1 | 0.15% | 0.3283 |
| map00950 | Isoquinoline alkaloid biosynthesis | 1 | 0.15% | 0.3283 |
| map00960 | Tropane, piperidine and pyridine alkaloid biosynthesis | 1 | 0.15% | 0.3283 |
| map00750 | Vitamin B6 metabolism | 1 | 0.15% | 0.3283 |
| map01120 | Microbial metabolism in diverse environments | 11 | 1.64% | 0.3405 |
| map00630 | Glyoxylate and dicarboxylate metabolism | 2 | 0.30% | 0.3448 |
| map04721 | Synaptic vesicle cycle | 2 | 0.30% | 0.3448 |
| map00350 | Tyrosine metabolism | 2 | 0.30% | 0.3653 |
| map04976 | Bile secretion | 1 | 0.15% | 0.3655 |
| map00310 | Lysine degradation | 1 | 0.15% | 0.3655 |
| map04330 | Notch signaling pathway | 1 | 0.15% | 0.3655 |
| map04962 | Vasopressin-regulated water reabsorption | 1 | 0.15% | 0.3655 |
| map00240 | Pyrimidine metabolism | 5 | 0.75% | 0.3765 |
| map03430 | Mismatch repair | 2 | 0.30% | 0.3855 |
| map04666 | Fc gamma R-mediated phagocytosis | 1 | 0.15% | 0.4006 |
| map00563 | Glycosylphosphatidylinositol(GPI)-anchor biosynthesis | 1 | 0.15% | 0.4006 |
| map03040 | Spliceosome | 6 | 0.89% | 0.4283 |
| map04210 | Apoptosis | 1 | 0.15% | 0.4339 |
| map00591 | Linoleic acid metabolism | 1 | 0.15% | 0.4339 |
| map00480 | Glutathione metabolism | 2 | 0.30% | 0.4634 |
| map03060 | Protein export | 2 | 0.30% | 0.4634 |
| map00592 | alpha-Linolenic acid metabolism | 1 | 0.15% | 0.4653 |
| map00906 | Carotenoid biosynthesis | 1 | 0.15% | 0.4653 |
| map00360 | Phenylalanine metabolism | 1 | 0.15% | 0.4653 |
| map00020 | Citrate cycle (TCA cycle) | 2 | 0.30% | 0.482 |
| map00100 | Steroid biosynthesis | 1 | 0.15% | 0.4949 |
| map04144 | Endocytosis | 2 | 0.30% | 0.5179 |
| map04145 | Phagosome | 2 | 0.30% | 0.5179 |
| map04115 | p53 signaling pathway | 1 | 0.15% | 0.523 |
| map03010 | Ribosome | 6 | 0.89% | 0.5565 |
| map01100 | Metabolic pathways | 40 | 5.96% | 0.5612 |
| map00860 | Porphyrin and chlorophyll metabolism | 2 | 0.30% | 0.5687 |
| map01040 | Biosynthesis of unsaturated fatty acids | 1 | 0.15% | 0.5745 |
| map00130 | Ubiquinone and other terpenoid-quinone biosynthesis | 1 | 0.15% | 0.5745 |
| map04130 | SNARE interactions in vesicular transport | 1 | 0.15% | 0.5982 |
| map03050 | Proteasome | 2 | 0.30% | 0.6004 |
| map00260 | Glycine, serine and threonine metabolism | 2 | 0.30% | 0.6156 |
| map00330 | Arginine and proline metabolism | 2 | 0.30% | 0.6446 |
| map00710 | Carbon fixation in photosynthetic organisms | 2 | 0.30% | 0.6446 |
| map03420 | Nucleotide excision repair | 2 | 0.30% | 0.6585 |
| map00561 | Glycerolipid metabolism | 1 | 0.15% | 0.6616 |
| map00980 | Metabolism of xenobiotics by cytochrome P450 | 1 | 0.15% | 0.6616 |
| map00190 | Oxidative phosphorylation | 3 | 0.45% | 0.6764 |
| map04310 | Wnt signaling pathway | 1 | 0.15% | 0.6805 |
| map03440 | Homologous recombination | 1 | 0.15% | 0.6983 |
| map00900 | Terpenoid backbone biosynthesis | 1 | 0.15% | 0.6983 |
| map00983 | Drug metabolism - other enzymes | 1 | 0.15% | 0.7151 |
| map03020 | RNA polymerase | 1 | 0.15% | 0.7151 |
| map04914 | Progesterone-mediated oocyte maturation | 1 | 0.15% | 0.731 |
| map00010 | Glycolysis / Gluconeogenesis | 2 | 0.30% | 0.7329 |
| map03460 | Fanconi anemia pathway | 1 | 0.15% | 0.7603 |
| map00250 | Alanine, aspartate and glutamate metabolism | 1 | 0.15% | 0.7863 |
| map00195 | Photosynthesis | 1 | 0.15% | 0.7863 |
| map03013 | RNA transport | 3 | 0.45% | 0.7878 |
| map04141 | Protein processing in endoplasmic reticulum | 2 | 0.30% | 0.9311 |
